# Supplementary material for: Antifungal Potential of Biogenic Zinc Oxide Nanoparticles for Controlling Cercospora Leaf Spot in Mung Bean
Source: Nanomaterials (Basel). 2025 Jan 19;15(2):143. doi: 10.3390/nano15020143 (PMC11767459; doi:10.3390/nano15020143)
Supplement: Supplementary file 1 [file nanomaterials-15-00143-s001.zip › nanomaterials-3394653-supplementary.pdf]

Table S1. The disease rating scale used for the measurement of Plant Disease Index (PDI)

Severity Rating	Symptoms on Plants	Status
0	No observable plant symptoms	Highly Resistant
1	1–20 % of the foliage or pod area has tiny pinhead-sized lesions.	Resistant
3	21-40 % of the foliage or pod area has tiny, brown spots on it.	Moderately Resistant
5	41–60% of the foliage or pod area has large spots on it.	Moderately Susceptible
7	61–80% of the foliage or pod area is damaged by larger coalescing spots.	Susceptible
9	81–100% of the foliage or pod area is affected by bigger consolidating spots.	Highly Susceptible

Table S2: The values of crystallite size *D* using Scherrer formula

$2\theta^o$	θ^o	Plane	FWHM	*D* (nm)
31.713	15.8565	(100)	0.0024979	60.26534
34.401	17.2005	(002)	0.0033115	45.77762
36.216	18.108	(101)	0.0042071	36.21452
47.533	23.7665	(102)	0.0074587	21.21478
56.556	28.278	(110)	0.0057192	28.7511
62.796	31.398	(103)	0.0040743	41.63996
67.938	33.969	(112)	0.0023123	75.51999
68.984	34.492	(201)	0.0030773	57.09726

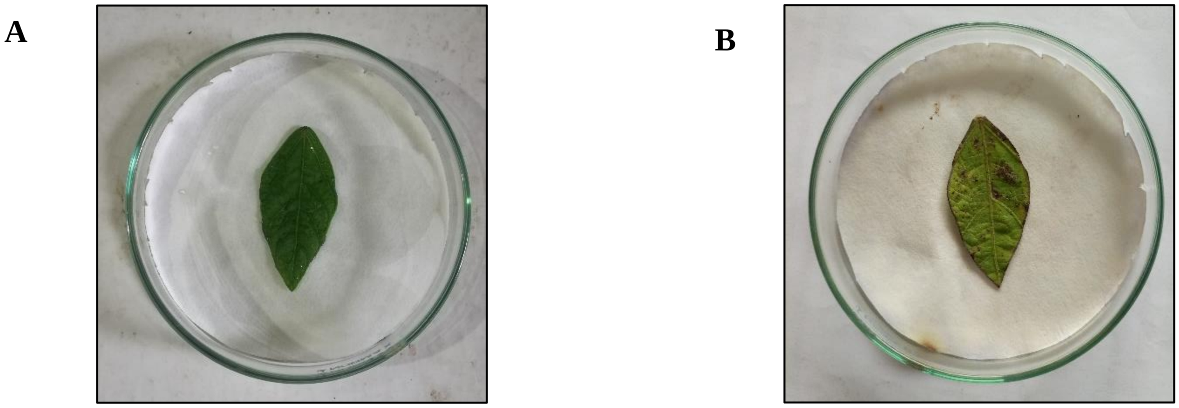

Figure S1. Disease symptoms caused by *C. canescens* on detached mungbean leaves. A= 1st day post inoculation. B= 9th day post inoculation.

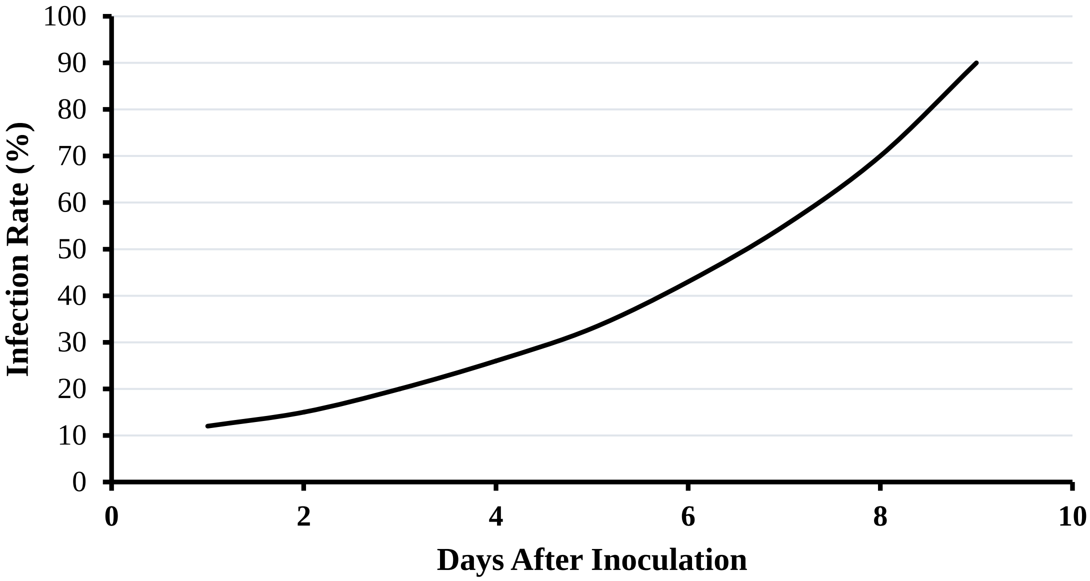

Figure S2. Cercospora leaf spot disease progression curve up till 9th day post inoculation.

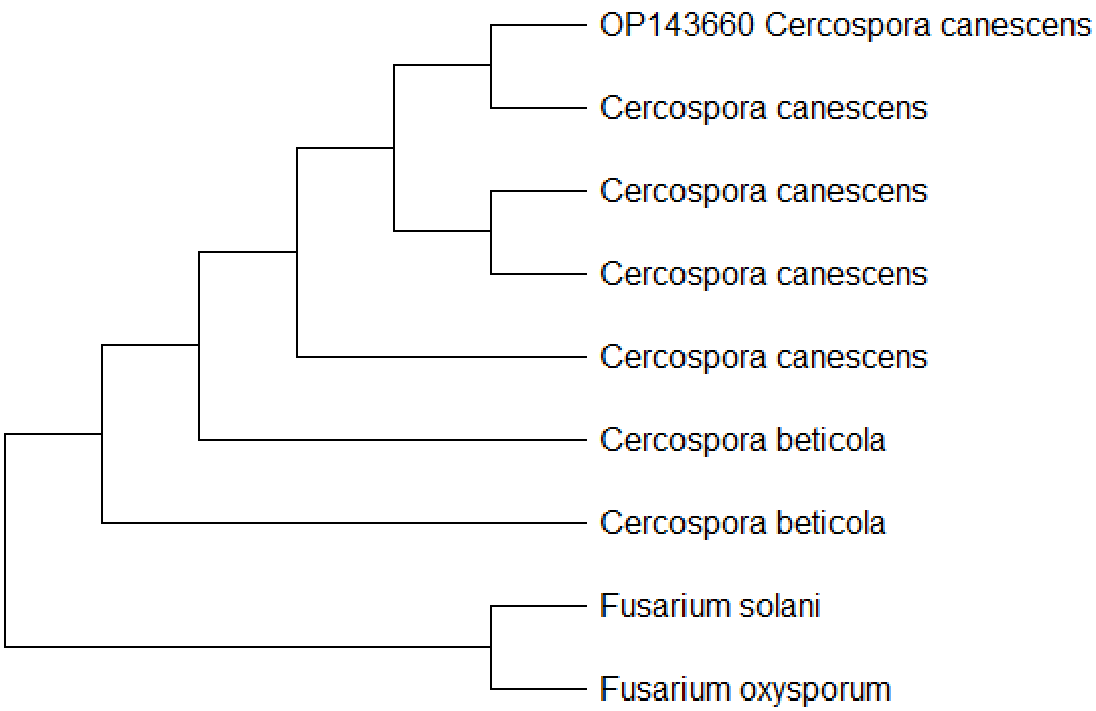

Figure S3. The phylogenetic tree of *C. canescens* isolates (OP143660) obtained in this study compared with ITS sequences.

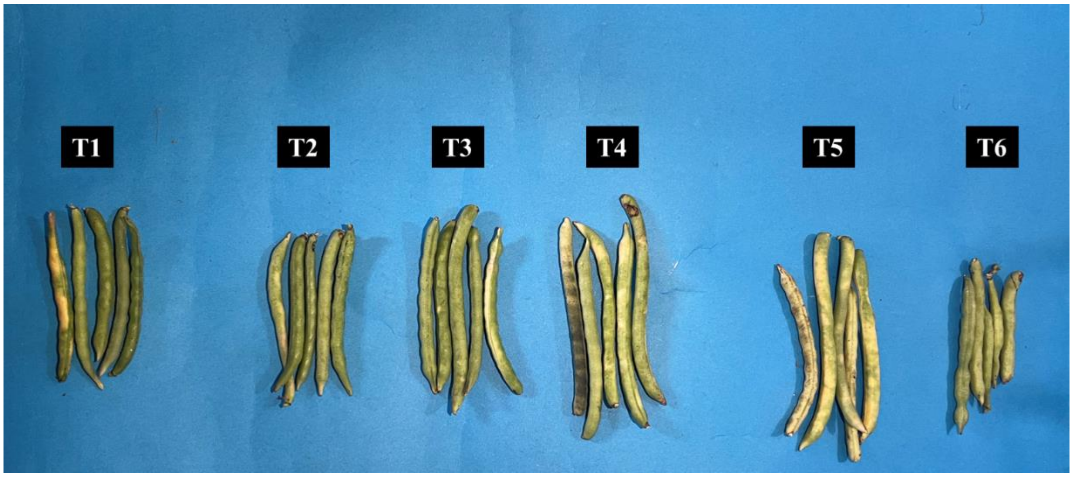

Figure S4. The effect of green synthesized ZnO NPs on mungbean pod size.

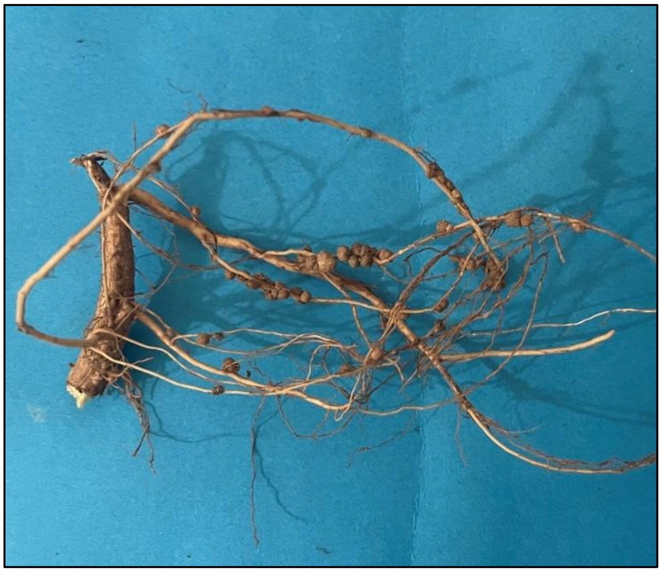

Supplemental Figure S5. Presence of root nodules in mungbean plants treated with ZnO NPs.
